# Influence of Daily Meteorological Changes on Stroke Incidence Across the United States

**DOI:** 10.5811/westjem.39685

**Published:** 2025-07-11

**Authors:** Randall L. Ung, Jeffrey S. Lubin

**Affiliations:** Penn State Milton S. Hershey Medical Center, Department of Emergency Medicine, Hershey, Pennsylvania

## Abstract

**Introduction:**

Various variables of weather are hypothesized to exert a small but measurable, significant influence on the development of cerebral infarctions (strokes). Improved characterization of this relationship would enhance understanding of the impact of climate change on healthcare demand. However, current data are conflicting regarding the exact nature of the direction and magnitude of the relationship between weather variables and stroke incidence.

**Methods:**

We conducted a retrospective analysis using patient data from 2019 across the contiguous United States obtained from the TriNetX global research data network and weather data from the National Oceanic and Atmospheric Administration database. Data from hospitalized patients who had a diagnosis of cerebral infarction, as defined from *International Classification of Diseases, 10^th^ Rev*, diagnosis codes, were used for analysis. Negative binomial regression calculated the incidence rate ratio (IRR) between stroke and various weather variables: temperature (°C), change in temperature, pressure, change in pressure, and precipitation.

**Results:**

Our study included 92,422 patients across 92 healthcare systems. Regression analysis revealed a small but statistically significant association between stroke and change in temperature (IRR 1.0047, confidence interval 1.0012 – 1.0083, *P* = .010). The remaining variables in our model did not have a statistically significant effect on incidence of stroke.

**Conclusion:**

The data suggest that one aspect of weather, specifically day-to-day increases of ambient temperature, has a measurable small magnitude but statistically significant impact on local stroke patterns.

## INTRODUCTION

Cerebral infarctions (strokes) are a leading cause of death and disability within the United States (US) and globally,^1^ and the incidence of strokes has been rising and is expected to continue to rise.^2^ The occurrence of stroke has been associated with several risk factors, including hypertension, smoking, and diabetes.^3^–^5^ The effect of weather on the incidence of stroke is less clear however.^6^ Geographic trends, such as increased rates of strokes in the southeast region of the US (often referred to as the “stroke belt”), suggest a possible link with weather.^7^,^8^ Previous studies have suggested that precipitation, temperature, and pressure effect changes in the incidence of strokes, yet their conclusions are conflicting.^9^–^12^ The exact role of these weather variables in the development of stroke, therefore, is controversial.

Evolving patterns in weather with climate change are inevitable, and these changes may influence the occurrence of stroke.^13^ Many areas of the US have experienced dramatic changes in temperature averages and precipitation in recent years; thus, a more comprehensive understanding of the relationship between weather and stroke could be an invaluable tool moving forward. This may aid in preparation and resource allocation for regions and periods at risk for increased cases of stroke. In this study, we incorporated various meteorological variables into a single statistical model to delineate how these factors may influence the incidence of stroke across the contiguous US.

### Materials and Methods

In this retrospective study we used criteria as outlined in Worster and Bledsoe to optimize the quality of our investigation.^14^ Specifically, we were trained on use of the databases to identify criteria for inclusion and exclusion, define variables used, define the database used, describe how datapoints were sampled/obtained, and outline management of missing data.

### Data Collection

We collected the data used in this study from the TriNetX Diamond Network, which provided access to electronic health records from approximately 212 million patients from 92 healthcare organizations. The retrospective study was reviewed and accepted by Penn State University’s Institutional Review Board (STUDY00022926 approved June 28, 2023) and determined to be exempt from informed consent.

Raw patient and encounter data was extracted from the TriNetX database for analysis on custom scripts in Python (Python Software Foundation, Wilmington, DE) and R (R Foundation for Statistical Computing, Vienna, Austria). (We did not use analytical tools within TriNetX). The study included hospitalized patients ≥ 18 years of age diagnosed with cerebral infarction (as defined using *International Classification of Diseases, 10^th^ Rev*. code I63). Only the first stroke diagnosis per hospital admission was counted, and admissions within seven days were treated as the same encounter to avoid multiple counts. Cerebral infarction did not need to be the primary diagnosis. Data included only patients within the contiguous US. We excluded regions lacking weather data for specific days or entirely and regions without patient data.

We used the data from the National Oceanic and Atmospheric Administration through Google Cloud’s BigQuery to obtain weather variables from weather stations across the contiguous US in 2019. US Census data from BigQuery were used to match the weather station location to the appropriate three-digit ZIP code. In three-digit ZIP code regions with multiple weather station data, we averaged data across all the stations within each region. Of note, in less populous areas of the US (particularly in the West), no ZIP code is defined, and these regions were excluded from analysis. Additionally, some three-digit ZIP code regions lacked weather data for particular days or altogether and were excluded from our analysis. Likewise, regions that lacked patient data from TriNetX were not included in the data. We created custom SQL scripts to obtain and organize weather data for the study.

Based on previous studies, we selected temperature, pressure, and amount of precipitation to use for our model. Additionally, the change in both temperature and pressure was calculated as the difference in the current weather value to the average of the previous seven days. We calculated the variance inflation factor to ensure these variables did not have significant multicollinearity, maintaining a value < 3. We filtered and merged patient and weather data using custom Python v3.11.5 scripts.

**Population Health Research Capsule**

What do we already know about this issue?

*Weather variables are hypothesized to exert a small but measurable significant influence on the development of cerebral infarctions (strokes)*.

What was the research question?

*At a national scale, how do weather variables influence the incidence of strokes within the United States?*

What was the major finding of the study?

*Increase in temperature is associated with increase in stroke incidence: IRR 1.0047, CI 1.0012 – 1.0083, P = .01*.

How does this improve population health?

*While these findings expand our understanding of weather and stroke relationship, they would not support varying emergency staffing given the small effect size*.

### Statistical Analysis

We used a negative binomial regression mixed-effects model to understand how weather variables affected the incidence of strokes per three-digit ZIP code. A mixed-effects model was used to control for the repeated measures in each three-digit ZIP code region. The incidence rate ratio (IRR) is reported for each weather variable. We scaled data before regression analysis, and regression coefficients were “unscaled” to calculate the incidence rate ratio (IRR) in relatable units. We implemented the Wald method for the calculation of 95% confidence intervals (CI). Statistical analysis was implemented using a combination of custom Python v3.11.5 and R v4.3.1 scripts.

## RESULTS

Our study included 92,422 patients across 349 three-digit ZIP code regions within the US during 2019. After matching with available weather data, 85,355 occurrences of strokes were included in our analysis. Across all three-digit ZIP codes, the average daily incidence of stroke in each region was 0.69 (SD 0.95). Characteristics of the patient population are outlined in Table 1. Summary statistics for ZIP code regions are outlined in Table 2. Negative binomial mixed-effects regression revealed notable variability in the relationship between weather variables and stroke among the different regions included in our dataset (Figure). The fixed effects from our regression analysis showed that among the weather variables we studied, only the change in temperature had a significant association with the incidence of stroke (Table 3). Specifically, our study showed that an increase in temperature (IRR 1.0047, CI 1.0012–1.0083, *P* = .01) was associated with a positive change in the incidence of stroke. The remaining variables did not have a significant impact on stroke incidence: temperature (IRR 1.0001, CI 0.9985–1.0017, *P* = .91), pressure (IRR 0.9999, CI 0.9961–1.0037, *P* = .94), change in pressure (IRR 1.0021, CI 0.9983–1.0059, *P* = .28), precipitation (IRR 0.9996, CI 0.9980–1.0012, *P* = .64).

## DISCUSSION

This study suggests that one variable characterizing daily weather patterns has a small but statistically significant impact on the incidence of strokes across the contiguous US. Specifically, increases in daily temperature from the prior seven-day average are associated with significant small but measurable increases in the incidence of strokes. Results suggest that for every 1°C increase of temperature, the rate of stroke is increased by 1.0047 times, or 0.47%. This translates to a 4.8% increase in stroke incidence for a 10°C increase in temperature. The magnitudes of daily temperature, pressure, changes in pressure, and precipitation were not associated with statistically significant changes in stroke incidence.

Our findings add to the current and somewhat conflicting literature regarding the effect of weather on stroke. Weather has been hypothesized to influence a variety of cardiovascular diseases including strokes. A recent study in Japan found that several cardiovascular diseases are similarly influenced by daily temperature changes, specifically that the daily range in temperature increases hospitalizations related to cardiovascular disease.^15^ This hypothesis extends to stroke.^6^,^16^ A recent multicenter study across 567 cities in 27 countries showed that deaths (vs incidence in our study) related to strokes and other cardiovascular diseases were increased in days with extreme temperatures.^17^ However, other studies have come to conflicting conclusions, complicating our understanding of this relationship. No consensus exists regarding the exact nature between meteorological variables and strokes.

Our data are consistent with data that suggest increased temperature and warmer seasons increase the incidence of stroke.^10^,^16^,^18^,^19^ These studies span multiple geographic locations with specific studies located in Scotland,^10^ Qatar,^18^ and the US,^19^ suggesting that this relationship may be generalizable across the globe and is not restricted to one specific geographic area or climate. On a cellular basis, multiple physiological mechanisms may drive this idea. One explanation is increased endothelial dysfunction with increased temperature.^20^ One study used flow-mediated dilation of the brachial artery as a proxy for endothelial function and found that warmer temperatures dampened the ability of the brachial artery to dilate in the setting of ischemia. Additionally, dehydration has been proposed as a possible mechanism for increased incidence of stroke considering its increased likelihood in the setting of warmer weather, but one study in Qatar failed to show this as a contributing factor.^18^ Blood pressure also changes with temperature,^21^ but this is unlikely to explain our finding considering that blood pressure decreases with warmer temperatures. A decrease in blood pressure would not likely be associated with an increased incidence of strokes.

However, many studies also show that stroke incidence increases with decreases in temperature as during winter months.^9^,^11^,^12^ Further, other studies suggest that both cold and hot weather can increase the occurrence of strokes with extreme temperatures in either direction being the driving force.^16^,^17^ Considering that extreme warm temperatures are likely associated with large changes in temperatures, this is not necessarily conflicting with our results. Lastly, the effect of temperature may depend on the type of stroke with one study showing that increases in temperature increased incidence of ischemic stroke but decreased the incidence of hemorrhagic stroke.^19^

Previous investigations have also shown that atmospheric pressure is associated with the development of strokes. However, our study failed to show an influence of atmospheric pressure and strokes. One study in the United States also failed to show a significant relationship between stroke and atmospheric pressure.^22^ Interestingly, this same study did show that decreases in atmospheric pressure were associated with increases in acute myocardial infarction suggesting weather may differentially affect different cardiovascular diseases. The notion that atmospheric pressure may not incur changes in stroke risk may explain the heterogeneity within the literature, with some studies showing a positive relationship^23^,^24^ and others showing the opposite.^10^,^18^

Precipitation has been the focus of fewer studies compared to temperature and pressure, and studies have also had conflicting conclusions on its relationship with stroke.^25^,^26^ In our study, precipitation did not statistically affect stroke incidence. Although precipitation does not appear to influence the incidence of strokes, one study found increased precipitation was associated with improved outcomes in patients admitted for stroke.^11^

Unfortunately, the culmination of multiple studies assessing weather and stroke has not provided a clear consensus on the exact nature of their relationship. Varying methodologies may explain the discrepancies among the many studies, including ours. For one, several limitations exist in prior studies diminishing the generalizability of their conclusions. Varying geographic regions and different timescales used between studies may explain some of the conflicting conclusions. For instance, results from studies within a single city or small geographic region may have a strong effect on local stroke incidence but are poorly generalizable. The regional variability observed in our study, with some areas showing an increase in stroke incidence and others showing a decrease (Figure), can be attributed to several factors. Geographic and climatic differences, population characteristics, and local behaviors may all play a role in modulating the impact of temperature changes on stroke incidence. For example, regions with more extreme temperature fluctuations may experience different effects compared to regions with more stable climates.

Additionally, studies that average weather variables over large timescales, such as seasons, may mask important weather dynamics that happen on the timescale of days. Further, many studies focus only on a single meteorological variable without considering complex weather dynamics. Therefore, incorporating these factors into a single framework may better delineate the relationship between stroke and weather in a more generalizable fashion. This is one of the main advantages that our study addresses. Our data cover a wide region, including diverse geographical and climate regions throughout the contiguous US. Additionally, the precision of our analysis is on the scale of days and incorporates multiple meteorological variables. Many previous studies did not have these advantage.^10^,^11^ However, some studies go even further showing that temperature may influence strokes at the timescale of hours,^19^ and this may provide a clearer picture of how weather drives the onset of stroke.

## LIMITATIONS

Our study is not without limitations. One of its limitations is that isolating the effects of meteorological variables is inherently difficult. Randomized control trials are not possible, and changes in the weather will undoubtedly cause changes in other health-related variables such as increased activity on more pleasant days leading to possible confounders. Our study is also limited to data from a single year. For greater generalizability, additional data spanning multiple years would be beneficial. This may further describe how climate change specifically has influenced this relationship between stroke and weather. Additional data on patient demographics may also highlight important socioeconomic factors.

## CONCLUSION

Our study suggests that increases in temperature have a positive association with stroke incidence. Our analysis benefits from multiple factors including a more precise timescale of days, more refined geographic regions at the three-digit ZIP code level, a diverse set of climate regions, and evaluation of multiple weather variables in a single model. While our study demonstrates a statistically significant association between temperature changes and stroke incidence, the effect size is small. Given that ischemic stroke patients constitute a small minority of patients seen daily in an emergency department, these findings would not support varying staffing based on temperature changes alone. The impact of temperature changes on stroke incidence, although measurable, is limited in its practical implications for emergency department operations and population health.

## Supplementary Information



## Figures and Tables

**Figure:**
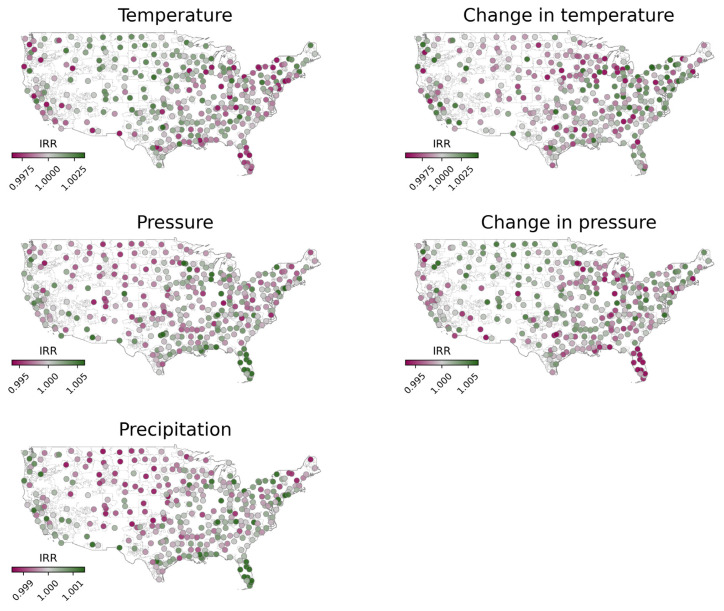
Incidence rate ratios for ZIP code regions as defined by random effects of negative binomial mixed-effects regression. Maps illustrate the relationship between weather variables and stroke. Each map depicts the IRR for each variable used in our regression model as a colored circle. Circles filled green depict a positive relationship between the variable and stroke incidence while magenta represents a negative association. Gray-filled circles have a weaker or zero association. The geography of ZIP code regions included in our analysis are outlined in gray. *IRR*, incidence rate ratio.

**Table 1:** Summary of patient statistics in a study of the association of weather on the incidence of stroke in the United States.

Total patients (N)	92,422
Age (median [IQR])	68 [59–77]
Sex (n [%])	
Female	46,465 [50.27 %]
Male	45,854 [49.61 %]
Unknown	4 [< 0.01 %]
Race (n)	
Black	7,080 [7.66 %]
Asian	262 [0.28 %]
White	20,372 [22.04 %]
Unknown	64,708 [70.01 %]

* The total number of patients corresponds to the incidence of strokes across hospital systems within the TriNetX database during 2019.

*IQR*, interquartile range.

**Table 2:** Summary of ZIP code region statistics in a study of the association of weather on the incidence of stroke in the United States.

Number of ZIP code regions (N)	349
Temperature (°C, mean ± SD)	13.2 ± 5.6
Pressure (hPa, mean ± SD)	1016.4 ± 1.4
Mean precipitation (mm, mean ± SD)	3.1 ± 1.2
Mean incidence of stroke (mean ± SD)	0.69 ± 0.95

* Incidence represents the mean number of strokes across all three-digit ZIP codes for hospital systems that are included in the database.

*hPa*, hectopascal; *mm*, millimeters.

**Table 3:** Summary of mixed-effects negative binomial regression in a study of the association of weather on the incidence of stroke in the United States.

Variable	IRR	CI	P
Temperature (°C)	1.0001	0.9984–1.0017	.91
Change in temperature (°C)	1.0047	1.0012–1.0083	.01*
Pressure (hPa)	0.9999	0.9961–1.0037	.94
Change in pressure (hPa)	1.0021	0.9983–1.0059	.28
Precipitation (mm)	0.9988	0.9980–1.0012	.64

*IRR*, incidence rate ratio; *CI*, confidence interval; *°C*, Celsius; *hPa*, hectopascal; *mm*, millimeters.
