# Supplementary figures and images for: Influence of Daily Meteorological Changes on Stroke Incidence Across the United States
Source: West J Emerg Med. 2025 Jul 11;26(4):984–9. doi: 10.5811/westjem.39685 (PMC12342417; doi:10.5811/westjem.39685)

### Temperature

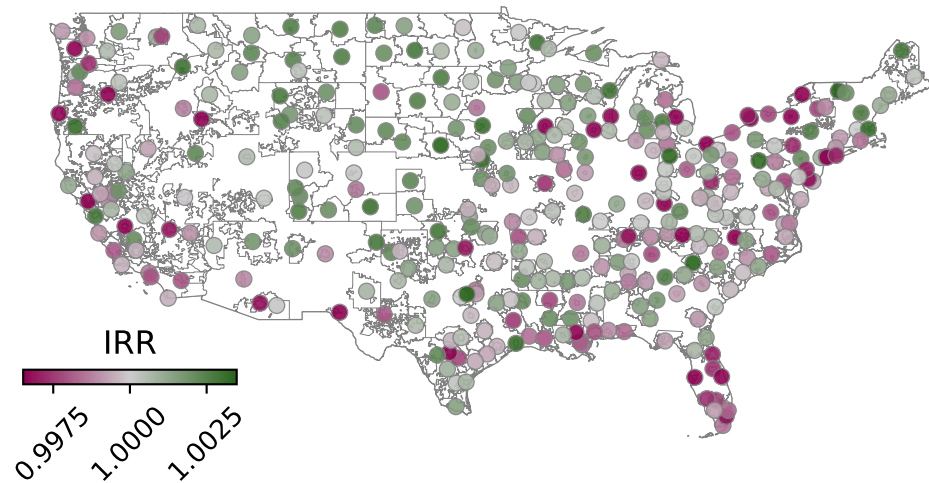

### Change in temperature

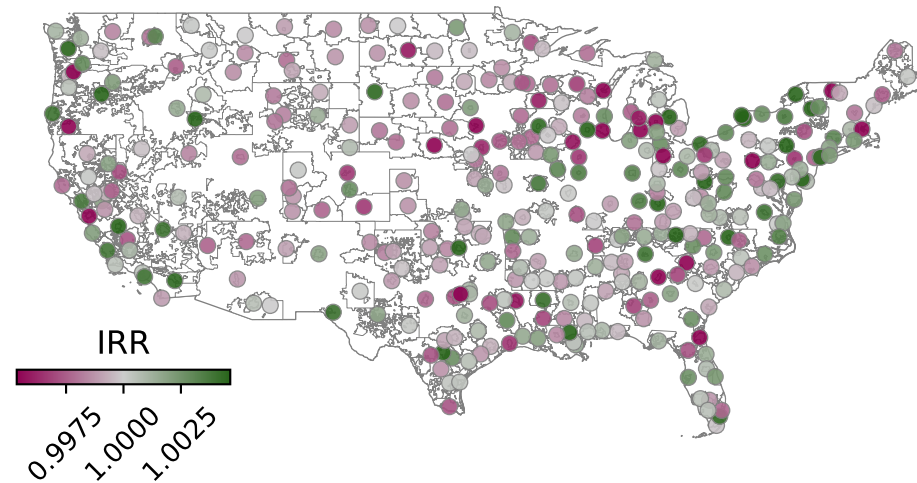

### Pressure

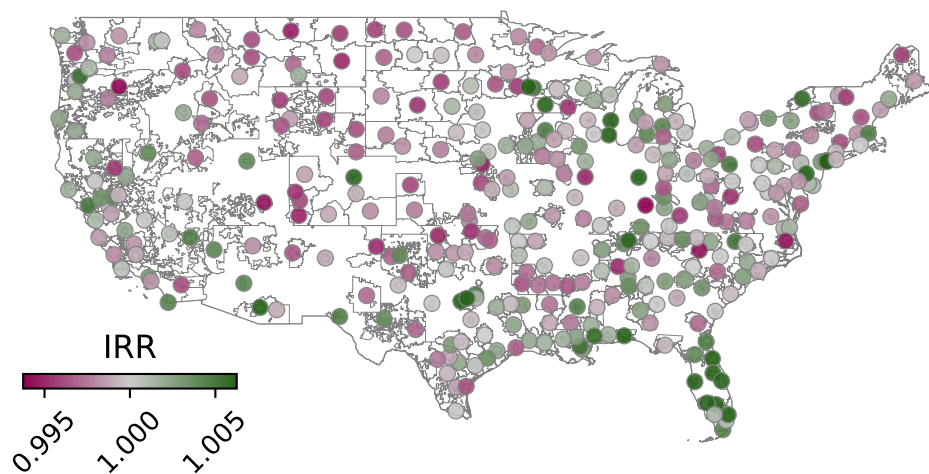

### Change in pressure

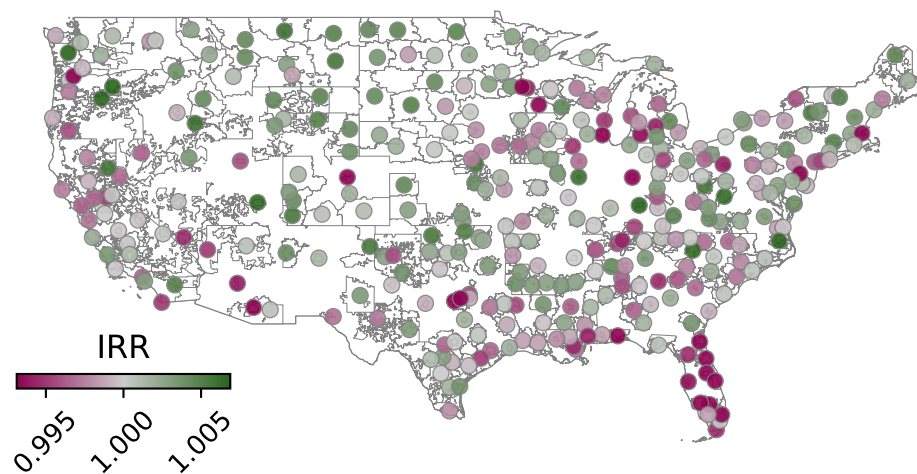

### Precipitation

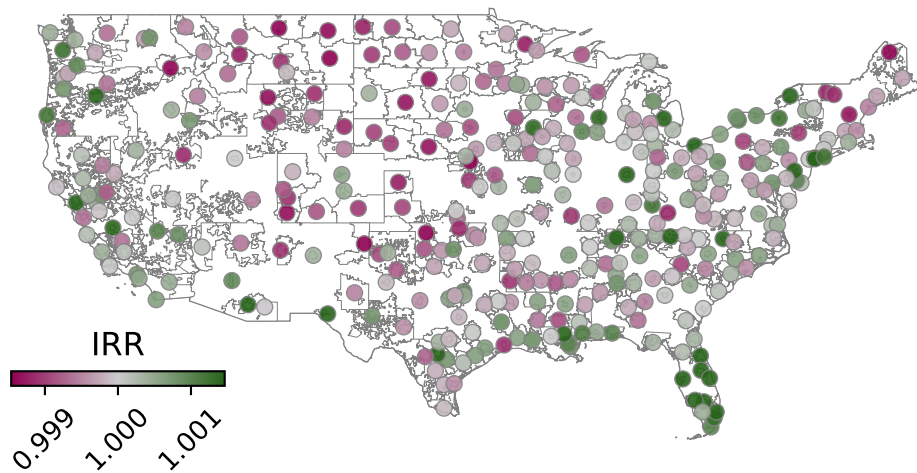

Supplement: Supplementary file 1 [file wjem-26-984-g001.pdf]
